# Parkinson's Disease: Impulsivity Does Not Cause Impulse Control Disorders but Boosts Their Severity

**DOI:** 10.3389/fpsyt.2018.00465

**Published:** 2018-09-28

**Authors:** Juan Marín-Lahoz, Javier Pagonabarraga, Saul Martinez-Horta, Ramón Fernandez de Bobadilla, Berta Pascual-Sedano, Jesús Pérez-Pérez, Alexandre Gironell, Jaime Kulisevsky

**Affiliations:** ^1^Movement Disorders Unit, Neurology Department, Hospital de la Santa Creu i Sant Pau, Barcelona, Spain; ^2^Biomedical Research Institute (IIB-Sant Pau), Barcelona, Spain; ^3^Centro de Investigación Biomédica en Red sobre Enfermedades Neurodegenerativas (CIBERNED), Madrid, Spain; ^4^Institute de Neurociencies Universitat Autonoma de Barcelona, Cerdanyola del Vallès, Spain; ^5^Centro Neurológico de Atención Integral, Navarra, Spain; ^6^Estudios de Ciencias de la Salud, Universitat Oberta de Catalunya, Barcelona, Spain

**Keywords:** impulsivity, impulse control disorders, behavioral addictions, Parkinson's disease, severity

## Abstract

**Introduction:** Impulse control disorders (ICDs) are a common complication of Parkinson's disease (PD) receiving dopamine agonist (DAA) Impulsivity is considered an underlying mechanism but evidence of this relationship is scarce. To explore the relationship between impulsivity and the presence and severity of ICD in PD.

**Methods:** Prospective cross-sectional study of consecutive PD outpatients. Patients with dementia or previously known ICDs were excluded. Two measures of impulsivity were assessed: Barratt Impulsiveness Scale (BIS-11) for impulsiveness trait (main exposure) and commission errors in the Continuous Performance Test (CE) for motor inhibition. Main outcomes were diagnosis of ICD based on a comprehensive clinical interview and severity of ICD based on the Questionnaire for Impulsive-Compulsive Disorders.

**Results:** Of 100 patients (mean [SD] age, 67.2 [8.8], 54 male), 31 had ICD. Patients with ICDs were 5.3 years younger (*p* = 0.01), used more frequently dopamine agonist (*p* = 0.02), alcohol (*p* = 0.009) and tobacco (*p* = 0.02). They were not more impulsive on BIS-11 (56 vs. 58, *p* = 0.23, adjusted *p* = 0.46) and CE (*p* = 0.96). No relationship was found between dopaminergic medications and impulsivity or ICD severity. Among patients with ICD, impulsivity was correlated with ICD severity (BIS-11 *r* = 0.33, *p* = 0.001, adjusted *p* = 0.002, CE *r* = 0.53, *p* = 0.006). Multivariate regression analysis confirmed the independent predictive role of both measures.

**Conclusions:** Impulsivity is not associated with increased prevalence of ICD in PD but it is strongly linked to ICD severity. When considering dopamine replacement therapy, assessment of impulsivity may be a useful approach to detect those patients at risk of severe forms of ICD.

## Introduction

Impulse control disorders (ICDs) are a common neuropsychiatric complication of Parkinson's disease (PD). By definition, ICDs refer to pathological behaviors characterized by failure to resist an impulse, drive or temptation to perform an act that is harmful. Usually, the affected individual experiences pleasure, gratification, or relief at the time of committing the act (1). Common ICDs in PD include dysfunctional behaviors related to gambling, sex, food intake, shopping, and hobbies. In the context of PD, these and other ICD-related behaviors are increasingly regarded as behavioral addictions (2–4).

ICDs are uncommon in the general population and in untreated PD patients (5–7). Among PD treatments, dopamine agonists (DAA) are strongly associated with ICDs (5). Patients receiving DAA for a disorder other than PD also have a high risk of ICDs (8, 9). However, other risk factors are important for their development and phenomenology as most patients taking DAA will not develop ICDs, and the best clinical-genetic predictive models for the development of ICD symptoms explains only part of the risk (4).

Not only ICD frequency is worrisome, but also for their range of severity. Severity may vary from extremely disruptive addictions causing bankruptcy, divorce, or even criminal prosecution (10, 11) to mild addictive symptoms—usually related to increased creativity or productivity—that may be even perceived as positive for patients' functionality (12, 13). It is also worth noting that 13–39% of patients with ICDs do not experience improvement or remission of the addictions after dopamine agonists withdrawal (3, 14–16). It is therefore important not only to study risk factors for the development of ICDs, but to identify the variables responsible for different prognosis and severity.

Impulsivity is a psychological construct characterized by poor control of thoughts and actions with a propensity to react fast over urges and environmental demands despite potential negative consequences. The definition of impulsivity shares obvious aspects with that of ICDs, and impulsivity is usually considered to have a causal relation with ICDs in PD. Higher scores on the Barratt Impulsiveness Scale (BIS-11), a self-reported questionnaire with semi-quantitative responses, have been observed in PD patients with and without ICDs (17, 18). Other modes of impulsivity, such as deficit of motor inhibition, have also been studied (18). Yet, studies on the influence of impulsivity for the development and clinical manifestations of ICDs in PD are scarce (17, 19).

In the present study, we aimed to explore the relation between impulsivity and frequency and severity of ICDs in PD. We assessed impulsivity and ICDs in PD patients who were taking dopaminergic drugs. Only incident cases of ICDs were included to avoid confounding factors such as changes in dopaminergic medication and a bias toward chronic ICDs. If impulsivity was a true risk factor for ICDs in PD we would expect higher levels of impulsivity in patients with ICDs.

## Materials and methods

Consecutive PD outpatients followed at the Movement Disorders Unit at Hospital de la Santa Creu i Sant Pau were invited to participate. Inclusion criteria were idiopathic PD, active treatment with a dopaminergic agent and last follow up at the same center including ICDs evaluation within the last 6 months. Exclusion criteria were: any other neurological condition, history of brain surgery, dementia according to Movement Disorder Society PD-dementia criteria (20), inability to perform the proposed tasks, use of dopamine antagonists, unstable medical or psychiatric conditions (depression and anxiety under effective and stable treatment were not excluded), and presence of ICD in the previous follow up. Excluding patients with previous ICDs was chosen to get measures of the exposures as close to ICD inception as possible and to avoid bias generated by medication changes and chronicity.

Patients were informed about the study during follow-up visits. If they agreed to participate in the study, they returned for the study evaluation. Patients were not excluded if ICDs were suspected or diagnosed the day they were informed about the study. In such cases, no changes in medication were made before the study protocol was completed (always within a week). All the participants were evaluated by a neurologist trained in movement disorders and a neuropsychologist experienced in PD. All the patients gave written informed consent and the study protocol was approved by the clinical research ethics committee at Hospital de la Santa Creu i Sant Pau. All the study was conducted according to the principles expressed in the Declaration of Helsinki. All the evaluations were performed on medication.

### ICDs diagnosis and rating

The presence of ICDs was assessed through a comprehensive clinical interview. ICDs were considered present when the related behavior was dysfunctional according to the components of addiction proposed by Brown (21) and modified by Griffiths (22). This model considers six components that comprise addiction regardless of the involvement of drug use: salience, mood modification, tolerance, withdrawal symptoms, conflict, and relapse. At least 4 of the 6 components needed to be present to consider a behavior as ICD. To diagnose a patient with more than one ICD, each ICD had to be unrelated to the others and considered dysfunctional.

To obtain a semiquantitative measure of severity in patients with a diagnosis of ICD, we used the short version of the questionnaire for impulsive-compulsive disorders in PD (QUIPs) (23) (score range 0–13), the Minnesota Impulsive Disorders Interview (MIDI) (24) (score range 0–56), and the number of different ICDs. The number of ICDs was based on the clinical interview. ICD diagnosis and QUIPs score were considered main outcomes and the other ICD related variables were considered exploratory. The investigator rating the main outcomes was blinded to impulsivity measures.

### Impulsivity evaluation

Impulsivity was evaluated using two different approaches, the BIS-11 and the PEBL Continuous Performance Test (PCPT). The BIS-11 was designed to assess the personality trait of impulsiveness (25). It has 30 self-reported items grouped in three subtraits: motor, attentional, and nonplanning. We used BIS-11 as a subjective estimator for impulsivity. The PCPT is a continuous performance task programmed in the Psychology Experiment Building Language (26) based on the Conners Continuous Performance Task II (27). The PCPT was designed to assess motor inhibition and sustained attention. Participants have to press a key in response to any capital letter (except X) that appears on the screen. At the same time, they must refrain from responding to lures (any X that appears). Targets are much more common than lures, creating a tendency to respond to lures, an inhibition failure. Commission error rate (CE)—failure to avoid responding to lures—was used as an indirect but objective measure of impulsivity (28). The investigator rating impulsivity measures was blinded to patient outcomes (presence and severity of ICDs).

### PD-related variables

We recorded the motor part of Unified Parkinson's Disease Rating Scale (UPDRS-III) (29), Hoehn and Yahr stage (H&Y), age at PD onset, PD duration, and current medical treatment. We calculated the Levodopa equivalent daily dose (LEDD) and the amount of LEDD corresponding to dopamine agonists (agonist-LEDD) according to previously reported conversion factors (30). We also recorded personal use of legal drugs (caffeine, nicotine and alcohol), use of illegal drugs and both personal and family history of alcohol and illegal drugs use disorders.

### Data analysis

Patients with and without ICD (ICD+/ ICD-) were compared. The chi-squared test was used for discrete variables. When this was inappropriate, the Fischer exact test was used. For most quantitative variables we used mean and Student's *t*-test and for those which did not comply with parametric assumptions we used median and Mann–Whitney *U*-test. We used Pearson correlations in ICD+ patients to analyze the relationship between ICD severity and other variables. Linear regression was used to assess whether impulsivity independently explained ICD severity and to control for motor state (UPDRS III) when comparing medication doses between groups. Logistic regression was used to assed which variables were independently associated with ICD diagnosis. The level of significance was set as *p* < 0.05, 2-sided. Main objectives were the comparison of BIS-11 score between ICD+ and ICD- and the correlation between BIS-11 with severity among ICD+ (measured by QUIPs). Multiple comparison adjustment was performed for these two tests by Bonferroni (using the number of tests performed for impulsivity and ICD diagnosis and for impulsivity and ICD severity). The other statistical analysis were considered exploratory and significance was not adjusted. Confidence intervals (CI) are reported at 95% level. All the statistical analyses were conducted using R version 3.1.3 (31).

## Results

### ICD frequency

One hundred consecutive PD patients (54 male, age mean ± SD 67 ± 9, education 11 ± 5, age at PD diagnosis 61 ± 9) were recruited and ICDs were diagnosed in 31. Thirty-eight patients had a positive score in QUIPs and 19 in MIDI.

The behaviors causing ICDs were hobbism/punding (*n* = 15), binge-eating (*n* = 14), pathological hypersexuality (*n* = 5), and compulsive shopping (*n* = 5). Hobbism/punding behaviors were tidying (*n* = 6), board games (*n* = 3), social networking (*n* = 3), repairing (*n* = 2), computer assisted edition (*n* = 2), and one case each of compulsive reading, doll handcraft, dancing, and walk-about. Eleven patients had several unrelated ICDs. None had pathological gambling or dopamine dysregulation syndrome.

ICD+ patients were 5.25 years younger at the time of the study (CI 1.24–9.25, *p* = 0.01) and at PD onset (*p* = 0.017). They were also more frequently receiving treatment with a DAA (59.4 vs. 83.9%, CI 4.7–44.1%, *p* = 0.02). No differences were found regarding time since PD diagnosis, Hoehn & Yahr stage, or UPDRS III status (Table 1).

**Table 1:** Clinical and behavioral description of the sample.

	**ICD- (*n* = 69)**	**ICD+ (*n* = 31)**	***p*-value**
Age, years	68.79 ± 7.82	63.55 ± 9.77	0.01
Gender, male (%)	37 (53.6)	17 (54.8)	0.91
Age at PD onset, years	62.5 ± 7.75	57.5 ± 9.52	0.02
Evolution, months	65.6 ± 3.76	77.8 ± 3.87	0.25
UPDRS III	23.4 ± 9.88	19.9 ± 9.65	0.11
H&Y^*^	2(2-2.5)	2(2-2)	0.06
LEDD, mg	533.1 ± 451.6	702.2 ± 416.9	0.07
Proportion of agonist use (%)	41 (59)	26 (84)	0.02
QUIPs	0.06	1.84	< 0.001
MIDI	0.29	3.3	< 0.001
BIS-11	56.35 ± 7.0	58.33 ± 7.72	0.23
Motor	19.31 ± 2.52	19.37 ± 2.95	0.93
Nonplanning	20.79 ± 4.07	22.23 ± 4.7	0.15
Attentional	16.25 ± 1.98	16.73 ± 1.98	0.27
PCPT commission errors ratio	0.378 ± 0.2	0.376 ± 0.2	0.96
PCPT correct RT, ms	480 ± 75	465 ± 57	0.24
PCPT error RT, ms	447 ± 163	426 ± 131	0.49
PCPT correct targets ratio	0.94	0.95	0.59
Current smokers (%)	3 (4.3)	6 (19)	0.02^**^
Lifetime smokers (%)	32 (46)	20 (65)	0.93
Age at smoking onset	19.65 ± 4.48	15.53 ± 3.11	< 0.001
Current alcohol users (%)	41 (59)	27 (87)	0.006
Lifetime alcohol users (%)	52 (75)	30 (97)	0.009
Lifetime illegal drugs users (%)	3 (5)	4 (14)	0.20^**^

*Mean ± standard deviation and proportions are shown unless otherwise specified. t-test is used for central measures and Chi-square for proportions unless other test specified*.

* *Median (interquartile range), Wilcoxon–Mann–Whitney test significance is shown*.

** *Fisher exact test significance is shown*.

*ICD, impulse control disorder; PD, Parkinson's disease; UPDRS, Unified Parkinson's Disease Rating Scale; H&Y Hoehn and Yahr scale; LEDD, levodopa equivalent daily dose; QUIPs, Short version of Questionnaire for Impulsive-Compulsive Disorders, MIDI, Minnesota Impulsive Disorders Interview; BIS-11, Barrat Impulsiveness Scale; PCPT, PEBL Continuous Performance Task; RT, reaction time; ms, milliseconds*.

Most patients in the sample were taking DAA: 41 used pramipexole, 11 ropinirole, and 15 rotigotine (among ICD+ patients 16, 4 and 6, respectively). No patients used more than one DAA. Average agonist-LEDD was higher in ICD+ patients (*p* = 0.012) but this difference was due to the higher proportion of DAA use in the ICD+ group (59.4% among ICD-, 83.9% among ICD+; chi-square test *p* = 0.02). Among patients taking DAA, no differences were found in agonist-LEDD between ICD+ and ICD- groups (*p* = 0.17). No difference was found after adjusting for UPDRS III (*p* = 0.22). Levodopa dose was similar in both groups (*p* = 0.35) and remained similar after adjusting for UPDRS III (*p* = 0.07). MAO-B inhibitors and amantadine use did not differ between groups (*p* = 0.96 and *p* = 0.22, respectively). LEDD showed a trend toward significance, with higher doses in ICD+ patients (*p* = 0.08). However, after controlling for UPDRS III this difference became clearly significant (*p* = 0.008), indicating that for a comparable degree of motor severity, ICD+ patients were taking higher LEDD.

No differences between ICD- and ICD+ groups were found either in BIS-11 (56.35 vs. 58.33, unadjusted *p* = 0.23, adjusted *p* = 0.46,) or in CE (*p* = 0.96) (Table 1). Neither BIS-11 nor CE, were related to LEDD, DAA use or agonist-LEDD (Table 2).

**Table 2:** Correlation matrix of impulsivity and dopaminergic medication.

	**BIS-11**	**CE**	**LEDD**	**Agonist-LEDD**
BIS-11		*r* = 0.03	*r* = 0.03	*r* = 0.03
CE	*p* = 0.76		*r* = 0.1	*r* = −0.12
LEDD	*p* = 0.77	*p* = 0.33		*r* = 0.43
Agonist-LEDD	*p* = 0.78	*p* = 0.22	*p* < 0.001	

*BIS-11, Barrat Impulsiveness Scale; CE, Commission Error rate in PEBL Continuous Performance Task; LEDD, Levodopa equivalent daily dose (mg/d), Agonist-LEDD, Levodopa equivalent daily dose corresponding to dopamine agonists(mg/d)*.

None of the patients had begun to consume legal or illegal drugs after PD diagnosis. No association was found between the current amount of alcohol intake and ICDs (*p* = 0.46). However, current alcohol use was associated to ICD (41.5 vs. 12.9%, OR = 5.42, CI = 1.64 - 23.61, *p* = 0.003), and patients with no history of alcohol use had a significantly lower prevalence of ICDs (6.7 vs. 34.5%, OR = 0.13, CI = 0.01-0.95, *p* = 0.03). Most of the participants did not remember the age of first alcohol use and therefore it was not analyzed.

Current tobacco use was associated with ICDs (OR = 5.17, CI = 1.05–34.44, *p* = 0.02), although < 10% of our sample were current smokers and the average tobacco consumption did not differ between groups (*p* = 0.25). Lifetime tobacco consumption was more common (52%) but its association with ICDs was not statistically significant (OR = 2.09, CI = 0.81–5.61, *p* = 0.09). However, patients who had begun smoking at 18 years old or younger were more likely to present ICDs than older first time smokers (OR = 7.18, CI 1.29–76.3, *p* = 0.01) and ICD+ patients had started smoking 4 years earlier on average (15.5 vs. 19.7 y.o., *p* < 0.001). Current and previous coffee intake was not related to ICDs (*p* = 0.73 and *p* = 0.67, respectively).

No patient was currently using illegal drugs and previous use was not significantly related to ICD diagnosis (OR = 3.26, CI = 0.51–23.87, *p* = 0.19). No association was found between ICDs and family history of drug or alcohol abuse (OR = 2.48, CI = 0.76–8.02, *p* = 0.08). Only one of the 18 patients who had never consumed alcohol on a regular basis and were not current smokers had ICDs (Fischer exact test *p* = 0.01).

Multiple logistic regression showed that only current alcohol consumption and age were independently associated with ICD diagnosis (Supplementary material).

### ICD severity

Among ICD+ we studied correlations patients between impulsivity measures and ICD severity measures. BIS-11 and QUIPs correlated significantly (*r* = 0.33, unadjusted *p* = 0.001, adjusted *p* = 0.002). We also found positive, significant correlations between each impulsivity measure and each severity measure, a correlation matrix is shown in Table 3. However, no correlation was found between BIS-11 and PCPT commission error rate (Pearson's *r* = 0.03, *p* = 0.75). Other variables related to ICD frequency were not statistically associated to severity measures (Supplementary Material).

**Table 3:** Correlation matrix of impulsivity and ICD severity among ICD+.

	**BIS-11**	**CE**	**QUIPs**	**MIDI**	**N. of ICDs**
BIS-11		*r* = 0.03	*r* = 0.33	*r* = 0.30	*r* = 0.30
CE	*p* = 0.75		*r* = 0.53	*r* = 0.38	*r* = 0.53
QUIPs	*p* = 0.001	*p* = 0.006		*r* = 0.68	*r* = 0.9
MIDI	*p* = 0.002	*p* = 0.04	*p* < 0.001		*r* = 0.70
N. of ICDs	*p* = 0.003	*p* = 0.006	*p* < 0.001	*p* < 0.001	

*ICD, impulsive control disorders; BIS-11, Barrat Impulsiveness Scale; CE, Commission Error rate in PEBL Continuous Performance Task; QUIPs, Short version of Questionnaire for Impulsive-Compulsive Disorders; MIDI, the Minnesota Impulsive Disorders Interview; N. of ICDs, number of ICDs*.

We performed multiple linear regression analysis to study whether each impulsivity estimator independently explained QUIPs in ICD+ patients. As the other tested variables were not statistically associated with QUIPs, we included as independent variables the ones that were associated with ICD presence except for age of smoking onset (because 32% of the ICD+ patients had never smoked) and history of alcohol use (because only one ICD+ patient had never used it). QUIPs score was the predicted value. We also used bidirectional stepwise regression to select the predictors. Both impulsivity measures—BIS-11 and commission error rate—significantly predicted QUIPs in the “all in” model. Current smoking was also a significant predictor. These were also the only variables selected by bidirectional stepwise regression (Table 4).

**Table 4:** Multiple linear regression models using QUIPs as the dependent variable among ICD+.

	**Adjusted *R*^2^**	**Estimate**	**t**	***p***
**All-in model**	0.5974			
(Intercept)		−3.55	−1.82	0.08
LEDD		0.00006	0.13	0.90
DAA use		0.055	0.11	0.91
Age		0.0007	0.037	0.97
BIS-11		0.065	2.45	0.02
CE		4.57	4.56	< 0.001
Current alcohol user		−0.459	−0.86	0.40
Current smoker		1.26	2.39	0.03
**Stepwise, both directions model**	0.6452			
(Intercept)		−3.52	−2.63	0.01
BIS-11		0.06	2.58	0.02
CE		4.35	4.82	< 0.001
Current Smoker		1.24	2.57	0.02

*QUIPs, Short version of Questionnaire for Impulsive-Compulsive Disorders; ICD, impulsive control disorders; LEDD, levodopa equivalent daily dose; DAA, dopamine agonists; BIS-11, Barrat Impulsiveness Scale; CE, Commission Errors*.

## Discussion

The present results show a complex interaction between DAA and impulsivity with the presence and severity of ICDs in PD patients. Contrary to our hypothesis impulsivity was not significantly higher in patients with ICD. However it was associated with higher severity of ICDs. This suggests that the role of impulsivity in ICD presence may be weak or nonexistent but it has an important role regulating ICD severity. The use of DAA, as previously shown, is associated with the presence of ICDs, but not with impulsivity or with ICD severity. Therefore DAA seem to have a critical role in ICD inception but not in their severity.

The fact that LEDD was higher in ICD+ only after controlling for motor severity might indicate that ICDs are related to an imbalance of dopaminergic activity between dorsal and ventral striatum. Higher doses of dopaminergic medication to control motor symptoms could promote overdosing of the ventral striatum (32), but our study is not designed to address this hypothesis.

Other studies have found higher levels of impulsivity in ICD+ patients (17). This discrepancy with our findings may be due to different study designs. Case-control studies select previously diagnosed patients who are more likely to have more severe ICDs (33). Systematic screening of consecutive patients permits to identify less conspicuous but relevant cases. Therefore, our sample, which excluded patients with previously known ICDs, is more likely to be enriched with less severe addictive behaviors. Both approaches are valid and useful to serve different purposes. The selection bias in case-control studies increases the probability to rule out suspected differences because the expected difference between groups is higher. Cross sectional studies such as the present study tend to produce more representative results. Another plausible explanation for the lack of significant differences in impulsivity between ICD+ and ICD- patients is based on the type of ICDs found in our sample. None of our patients had pathological gambling, while other studies analyzing risk factors for ICDs in PD included almost exclusively patients with gambling, a condition known to be highly related to elevated impulsivity (34).

The double dissociation exhibited by impulsivity and dopamine agonist use suggests that dopamine agonist do not cause PD-ICDs by means of fostering impulsivity. Impulsivity promotes the expression of the disorder not restraining the behaviors that constitute it. Conversely, the existence of an impulse able to constitute a disorder, would be caused by the imbalance generated by dopaminergic medication in the reward system (35). Accordingly, impulsive behaviors not generally linked to reward, such as reckless driving or domestic violence are quite rare in PD patients. Nonetheless, a minimum grade of impulsivity may be required as a perfect self-control would preclude any addictive behavior.

As previously reported (5), we found the use of legal drugs are greatly associated to ICDs. Drug use is linked to both reward imbalance and impulsivity, not shedding light on the discussed dissociation. In this sample, alcohol consumption was associated with the presence while tobacco was associated with both presence and severity of ICDs. The use of both drugs precedes ICDs development in this study, therefore they act as risk factors. History of alcohol and tobacco use is easily available information and probably not usually taken into account prior to DAA prescription as it has not been used in predictive models (4).

Our study has several limitations and strengths. The first limitation is that QUIP short has not been properly validated as a measure of ICD severity. However, it is considered sensitive, reliable, easy to answer accurately and able to capture ICD severity (23). A rating scale has been validated for ICD severity, the QUIP rating scale (36). This scale is closely related to QUIP short. To overcome this limitation, MIDI and the number of ICDs have also been studied yielding similar statistical correlations. Second, the design does not probe that impulsivity antecedes ICDs. Prospective studies are necessary to confirm causality. The strengths of our study are: (1) the assessment of two unrelated modes of impulsivity, showing that they were independently related to ICD severity; (2) the use of behavioral addiction criteria to diagnose ICDs, allowing the use of the same criteria independently of the studied behavior; and (3) a sample of systematically interviewed consecutive outpatients, avoiding selection bias.

## Conclusions

In summary, we found impulsivity is more associated with severity of ICDs than with ICD diagnosis. Conversely, we found DAA are associated the diagnosis of ICDs but are not associated with their severity. We also show how previous and current use of legal drugs is strongly related to the appearance and severity of ICDs. Further research is needed to evaluate whether impulsivity and legal drug use should be taken into account before prescribing DAA, and whether treatment strategies focused on decreasing impulsivity (37) in PD patients with ICDs would help to control these behaviors.

## Author contributions

JM-L conception, design, data collection, data analysis, data interpretation, writing, and editing. JP and JK design, data collection, data interpretation, writing, and editing. RF design and data collection. SM-H data interpretation, writing, and editing. BP-S, AG, and JP-P data collection and editing.

### Conflict of interest statement

The authors declare that the research was conducted in the absence of any commercial or financial relationships that could be construed as a potential conflict of interest.
