# Supplementary material for: Parkinson's Disease: Impulsivity Does Not Cause Impulse Control Disorders but Boosts Their Severity
Source: Front Psychiatry. 2018 Sep 28;9:465. doi: 10.3389/fpsyt.2018.00465 (PMC6172299; doi:10.3389/fpsyt.2018.00465)
Supplement: Supplementary file 1 [file Table_1.DOCX]

**Supplementary Materials 1.**

*ICD diagnosis logistic regression*

All the variables related to ICD diagnosis in the bivariate analysis were introduced into a logistic regression model to assess which ones were independently associated to ICDs. Current al use and age were the only significantly associated while current tobacco use and DAA use were not significantly associated. Bidirectional stepwise selection did not reject any variable.

Table S1: Multiple logistic regression models using ICD diagnosis as the dependent variable

|  | AIC | Estimate | z | p |
| --- | --- | --- | --- | --- |
| **All-in model** | 112.01 |  |  |  |
| (Intercept) |  | 1.12 | .52 | .601 |
| DAA use |  | 1.06 | 1.79 | .074 |
| Age |  | -0.57 | -1.96 | .049 |
| Current smoker |  | 1.38 | 1.71 | .088 |
| Current alcohol user |  | 1.31 | 2.01 | .036 |
| **Stepwise, both directions model** | 112.01 |  |  |  |
| (Intercept) |  | 1.12 | .52 | .601 |
| DAA use |  | 1.06 | 1.79 | .074 |
| Age |  | -0.57 | -1.96 | .049 |
| Current smoker |  | 1.38 | 1.71 | .088 |
| Current alcohol user |  | 1.31 | 2.01 | .036 |

Abbreviations: ICD=impulsive control disorders; AIC=Akaike information criterion, DAA=dopamine agonists.
