# Supplementary material for: Parkinson's Disease: Impulsivity Does Not Cause Impulse Control Disorders but Boosts Their Severity
Source: Front Psychiatry. 2018 Sep 28;9:465. doi: 10.3389/fpsyt.2018.00465 (PMC6172299; doi:10.3389/fpsyt.2018.00465)
Supplement: Supplementary file 2 [file Table_2.DOCX]

**Supplementary Materials 2.**

*Associations with ICD severity*

Although the main goal was to study the role of impulsivity, to further explore the possible explanatory variables of ICD severity we performed the Pearson correlations MIDI, QUIPs and the number of different ICD with of each factor associated with the ICD diagnosis.

Table S2: Correlation matrix of ICD severity estimators

|  | LEDD | Agonist LEDD | P. Smoking | C. Smoking | P. Alcohol | C. Alcohol | Age | Age Tabaco | QUIPs | MIDI | N. of ICDs |
| --- | --- | --- | --- | --- | --- | --- | --- | --- | --- | --- | --- |
| LEDD |  | r=0.5 | r=-0.16 | r=-0.18 | r=-0.01 | r=0.06 | r=0.15 | r=0.46 | r=0.03 | r=-0.15 | r=0.12 |
| Agonist LEDD | p<0.01 |  | r=-0.06 | r=0.09 | r=-0.12 | r=0.02 | r=0.02 | r=0.71 | r=0.17 | r=0.12 | r=0.14 |
| P. Smoking | p=0.38 | p=0.76 |  | r=0.31 | r=0.23 | r=0.27 | r=-0.28 | r=-0.84 | r=-0.08 | r=0.15 | r=-0.06 |
| C. Smoking | p=0.34 | p=0.63 | p=0.09 |  | r=0.45 | r=-0.11 | r=-0.09 | r=0.03 | r=0.3 | r=0.38 | r=0.18 |
| P. Alcohol | p=0.97 | p=0.52 | p=0.22 | p=0.01 |  | r=0.35 | r=0.1 | r=-0.15 | r=-0.01 | r=0.17 | r=-0.06 |
| C. Alcohol | p=0.73 | p=0.9 | p=0.14 | p=0.57 | p=0.06 |  | r=-0.05 | r=0 | r=-0.2 | r=-0.26 | r=-0.18 |
| Age | p=0.41 | p=0.94 | p=0.13 | p=0.64 | p=0.62 | p=0.81 |  | r=-0.26 | r=-0.02 | r=-0.05 | r=-0.06 |
| Age Smoking | p=0.25 | p=0.05 | p=0.01 | p=0.94 | p=0.75 | p=1 | p=0.53 |  | r=-0.01 | r=0.01 | r=0 |
| QUIPs | p=0.89 | p=0.37 | p=0.67 | p=0.1 | p=0.97 | p=0.28 | p=0.92 | p=0.99 |  | r=0.68 | r=0.9 |
| MIDI | p=0.42 | p=0.54 | p=0.42 | p=0.04 | p=0.38 | p=0.16 | p=0.79 | p=0.98 | p<0.01 |  | r=0.7 |
| N. of ICDs | p=0.52 | p=0.47 | p=0.76 | p=0.33 | p=0.77 | p=0.32 | p=0.75 | p=0.99 | p<0.01 | p<0.01 |  |

Abbreviations: ICD=impulsive control disorders; LEDD=levodopa equivalent daily dose; Agonist LEDD= LEDD corresponding to dopamine agonists; P. Smoking= previous smoking; C. Smoking= current smoking; P. Alcohol= previous alcohol consumption; C. Alcohol = current alcohol consumption; Age Smoking= Age begun to smoke; QUIPs= Short version of Questionnaire for Impulsive-Compulsive Disorders; MIDI N. of ICDs=number of ICDs.
